# Contribution of lung function in predicting distance covered in the 6-min walk test in obese Brazilian women

**DOI:** 10.1590/1414-431X202010279

**Published:** 2020-10-21

**Authors:** C.A. Luchesa, T.T. Mafort, R.R. Silva, I.C. Paro, F.M. Souza, A.J. Lopes

**Affiliations:** 1 Programa de Pós-Graduação em Ciências da Reabilitação, Centro Universitário Augusto Motta, Rio de Janeiro, RJ, Brasil; 2 Centro de Reabilitações, Centro Universitário Fundação Assis Gurgacz, Cascavel, PR, Brasil; 3 Programa de Pós-Graduação em Ciências Médicas, Faculdade de Ciências Médicas, Universidade do Estado do Rio de Janeiro, Rio de Janeiro, RJ, Brasil

**Keywords:** Obesity, Functional capacity, Exercise, 6-min walk test, Pulmonary function test

## Abstract

Obesity affects the respiratory system through various mechanisms, including systemic inflammation and direct mechanical hindrance due to fat deposition in the chest and abdomen. In addition, changes in the neural control of respiration and increases in thoracic blood volume can promote abnormalities in lung function. Thus, determining relationships between the distance covered in the 6-min walk test (6MWT) and demographic and lung function variables may help us better understand the mechanisms involved in reduced functional exercise capacity in obesity. To explore the determinants of the 6-min walking distance (6MWD) and evaluate the influence of lung function on the distance covered, 263 obese Brazilian women performed the 6MWT and underwent spirometry and respiratory muscle strength measurement. The mean age was 41.8±11.1 years. The mean body mass index (BMI) was 45±8 kg/m^2^. The 6MWD showed correlations with height (r=0.319), age (r=-0.281), weight (r=-0.370), BMI (r=-0.561), forced vital capacity (FVC, r=0.443), expiratory peak flow (r=0.278), maximal inspiratory pressure (MIP, r=0.326), and maximal expiratory pressure (r=0.259), all with P<0.0001. In the stepwise forward regression analysis, BMI, FVC, age, and MIP were the independent predictive variables for 6MWD, explaining 41% of its variability. The reference equation including lung function was as follows: 6MWD (m) = 513.6 - (4.439 × BMI_kg/m2_) + (1.136 × FVC_%predicted_) - (1.048 × age_yrs_) + (0.544 × MIP_%predicted_). Thus, the inclusion of lung function in a reference equation for 6MWD contributes to a better prediction of the distance covered in this population.

## Introduction

Obesity is a global public health problem, not only in developed countries but throughout the world (1). The prevalence of obesity has increased rapidly in the last 2 decades, and its presence is associated with significant morbidity, premature mortality, worse health-related quality of life (HRQoL), and higher health care costs (2). An increase in body weight has an effect not only on appearance, self-esteem, and social relationships but also on the level of health and, consequently, on the general condition and efficiency of the body (3). In addition, an increased amount of adipose tissue reduces muscle mass and strength, which limits the body's ability to maintain a prolonged effort without feeling fatigue (3).

The ability to walk a certain distance is an important measure of physical function and a valuable component of HRQoL because it reflects the ability to perform activities of daily living (ADLs) (4). In obese subjects, the lower skeletal muscle strength, cardiopulmonary capacity, and effort tolerance and the high metabolic costs translate to increased walking inefficiency, which together with the increased prevalence of associated comorbidities can impair walking (4). In addition, pain in overloaded joints during walking is a frequent complaint in obese individuals, who tend to walk more slowly and report dyspnea more frequently than non-obese individuals (5). Walking is the most accessible exercise modality for weight control. Performance tests, such as the 6-min walk test (6MWT), may reveal the limitations in the cardiorespiratory and motor functions underlying obesity-related disability (4,6). In this sense, determining the relationship between the 6-min walking distance (6MWD) and the demographic and functional variables of obese subjects may help to better understand the mechanisms involved in their reduced functional exercise capacity.

Some variables, such as age, anthropometric data, body composition, muscle strength, and disability, seem to have different degrees of impact on the 6MWD of obese individuals (4,6,7). Despite the importance of evaluating the performance of obese subjects during the 6MWT, few studies have analyzed the correlates of 6MWD in obese subjects. Moreover, although the results have been found to be highly reproducible, they are influenced by a number of factors, including the degree of obesity and aerobic capacity (7,8). To our knowledge, no previous study has evaluated the influence of lung function in predictive models for obese Brazilian individuals, despite the knowledge of the impact of obesity on lung function.

Obesity affects the respiratory system through several mechanisms, including systemic inflammation and direct mechanical changes due to fat deposition in the chest and abdomen (9). It increases respiratory work and therefore increases the neural respiratory drive, in addition to causing respiratory sleep disorders and, eventually, hypercapnic respiratory failure (10). In this context, pulmonary function tests may be useful to assess whether a physiological change can be explained by the effects of obesity on the respiratory system. An increase in the volume of adipose tissue in the perithoracic and abdominal regions reduces the compliance of the thoracic cavity and lung volumes and impairs diaphragmatic function, making the respiratory muscles work harder (11). The overall effect is an overload of the respiratory muscles, which increases respiratory effort, peak oxygen uptake (peak VO_2_), and energy expenditure (10).

According to the predictive equations in the literature for the 6MWT, obese individuals walk a shorter distance while working harder than non-obese people (4,7,12). The reference values obtained from healthy and normal-weight populations disregard the reduced performance capacity of obese individuals. Instead, specific reference values for this population can serve as a reference to assess the functional capacity at baseline, prescribe the appropriate exercise intensity, and monitor changes after rehabilitation interventions (4,13). Thus, the objectives of the present study were to further explore the determinants of the 6MWD and to assess the influence of lung function measurements on the 6MWD in Brazilian obese women.

## Material and Methods

### Patients

This was a cross-sectional study that evaluated women with body mass index (BMI) ≥30 kg/m^2^ and age ≥18 years who were in the preoperative period of bariatric surgery at Centro Universitário Fundação Assis Gurgacz (FAG), located in the city of Cascavel, Brazil. Those who met any of the following criteria were excluded: smokers or ex-smokers with a smoking status >10 pack-years; history of cardiac disease (including cardiac arrhythmia, unstable angina, myocardial infarction, uncontrolled hypertension [higher than 180/100 mmHg]); history of pleuropulmonary disease (including chronic obstructive pulmonary disease, asthma, and restrictive disorders other than obesity); orthopedic or neurological conditions that could cause changes in gait; prior hip or lower limb surgery; inability to perform pulmonary function tests; and inability to perform the 6MWT. Pathologies were confirmed using anamnesis, physical examination, and laboratory tests, when necessary. To define the patient's physical activity level, the International Physical Activity Questionnaire (IPAQ) was applied (14). All assessments were performed on the same day.

The protocol was approved by the Research Ethics Committee of FAG (CAAE No. 11613219.0.0000.5219), and all participants signed the informed consent form.

### Measurements

#### Anthropometry

Weight and height were measured using standard techniques, with participants wearing light clothing and no shoes. Weight was measured to the nearest 0.1 kg on a calibrated scale, and height was measured to the nearest 0.1 cm using a stadiometer. BMI was calculated as the weight in kilograms divided by the square of the height in meters (kg/m^2^), and the participants were classified according to the degree of obesity as follows: class I (BMI 30 to 34.9 kg/m^2^), class II (BMI 35 to 39.9 kg/m^2^), and class III (BMI ≥40 kg/m^2^) (1).

#### Lung function

Spirometry was performed using a MicroLoop portable spirometer (ML3535, Micro Medical, UK), while respiratory muscle strength was measured using a GlobalMed digital manometer (MVD 300, Brazil). The results of these tests are reported as the percentage of the predicted value (15,16).

#### 6MWT

Functional exercise capacity was evaluated using the 6MWT according to the guidelines of the American Thoracic Society (17). The test was performed in a 30-m corridor with a hard, flat surface marked every 3 m with colored tape on the floor and with 2 cones indicating the length of the walkway. Before and at the end of the test, the heart rate (Δ HR), systolic blood pressure (Δ SBP), diastolic blood pressure (Δ DBP), peripheral oxygen saturation (SpO_2_) and Borg's Perceived Exertion Scale (BPES) score were measured. The BPES was applied at baseline and at 6 min of the 6MWT using a range from 0 (nothing at all) to 10 (extremely severe) (18). Participants were instructed to walk as fast as possible. They were allowed to stop or rest during the test, if necessary. Two tests were performed with a minimum interval of 30 min of rest to avoid the effect of learning and adaptation, and the highest 6MWD was used for analysis. The 6MWT has been shown to be valid and reliable in obese individuals (8).

### Statistical analysis

Data were analyzed using SAS 6.11 (SAS Institute, Inc., USA). The results are reported as means±SD or frequency (percentage). The normality of data distribution was assessed using the Kolmogorov-Smirnov test and visual analysis of histograms. The association of 6MWD with demographic and anthropometric and lung function data was determined by the Pearson correlation coefficient (r). Stepwise forward linear regression analysis was applied to identify the demographic, anthropometric, and functional independent variables explaining the 6MWD. Two regression models were estimated in this study. In the first model, only the demographic and anthropometric data (basic model) were the independent variables. In the second model, in addition to clinical data, pulmonary function parameters were also included as independent variables (final model). The backward stepwise method was performed to select independent variables in the multiple linear regression models, and only variables with P<0.10 in the bivariate analysis were retained in the basic and final models.

Calibration was verified using the calibration plot (6MWD observed *vs* predicted, along with regression lines showing the slope and intercept) and the limits-of-agreement (LoA) plot by Bland-Altman method. Additionally, we used one-way analysis of variance with Tukey's multiple comparison test to find any significant differences in clinical or lung function variables among the 3 obesity classes. P<0.05 was considered statistically significant.

## Results

Of the 302 obese women eligible for the study, 39 were excluded for the following reasons: history of smoking >10 packs-year (n=21), history of cardiopulmonary disease (n=10), history of orthopedic or neurological disease (n=5), and reported prior hip or lower limb surgery (n=3). Thus, the evaluated sample consisted of 263 obese women.

The mean age was 41.8±11.1 years, while the mean BMI was 45±8 kg/m^2^. In terms of the level of physical activity assessed by the IPAQ, 172 patients were sedentary, 77 were insufficiently active, and 14 were active; none were considered “very active”. Twenty-eight patients were taking beta-blockers, 24 were taking statins, and 17 were taking oral hypoglycemic agents.

The mean 6MWD was 428.3±85.7 m; 86 (32.7%) participants walked <400 m, and 177 (67.3%) participants walked ≥400 m. Regarding lung function, 84 (31.9%) and 49 (18.6%) participants had forced vital capacity (FVC) and peak expiratory flow (PEF) <80% of predicted, respectively. Maximal inspiratory pressure (MIP) and maximal expiratory pressure (MEP) were <80% of predicted in 54 (20.5%) and 37 (14.1%) participants, respectively. Demographic data, metabolic status, comorbidities, lung function, and functional capacity are listed in Table 1.

**Table 1:** Demographic and anthropometric parameters, nutritional status, lung function, and functional capacity of obese women.

Variables	
Demographic and anthropometric data	
Age (years)	41.8±11.1
Weight (kg)	116.2±20.6
Height (cm)	161±0.07
BMI (kg/m^2^)	45±8
Obesity classification	
Class 1 (%)	13 (4.94)
Class 2 (%)	56 (21.3)
Class 3 (%)	194 (73.8)
Comorbidities	
Hypertension (%)	54 (20.5)
Dyslipidemia (%)	33 (12.5)
Type 2 diabetes (%)	25 (9.50)
Lung function	
FVC (% predicted)	88.4±17.7
PEF (% predicted)	99.2±22
MIP (% predicted)	106.4±21.4
MEP (% predicted)	96.4±21.7
6-min test distance	
6MWD (m)	428.3±85.7
Δ BPES	3.65±1.83
Δ HR (bpm)	22±6.75
Δ SBP (mm Hg)	19±8.43
Δ DBP (mm Hg)	17±9.50
Δ SpO_2_ (%)	-1.53±2.16

Data are reported as means±SD or frequencies (%). BMI: body mass index; FVC: forced vital capacity; PEF: peak expiratory flow; MIP: maximal inspiratory pressure; MEP: maximal expiratory pressure; 6MWD: 6-min walk distance; Δ BPES: difference in Borg's Perceived Exertion Scale between 0' and 6'; Δ HR: difference in heart rate between 0' and 6'; Δ SBP: difference in systolic blood pressure between 0' and 6'; Δ DBP: difference in diastolic blood pressure between 0' and 6'; Δ SpO_2_: difference in peripheral oxygen saturation between 0' and 6'.

We compared the data on lung function and functional capacity among the 3 obesity classes. There were significant differences in FVC values (class I = 104.8±14 *vs* class II = 89.2±16.6 *vs* class III = 87±17.4% of predicted), with class I significantly different from class II (P<0.01) and class III (P<0.001). There were significant differences in 6MWD values (class I = 585.8±112.9 *vs* class II = 430.1±73.8 *vs* class III = 417.3±76.4 m), with class I significantly different from class II (P<0.0001) and class III (P<0.0001).

**Figure 1:**
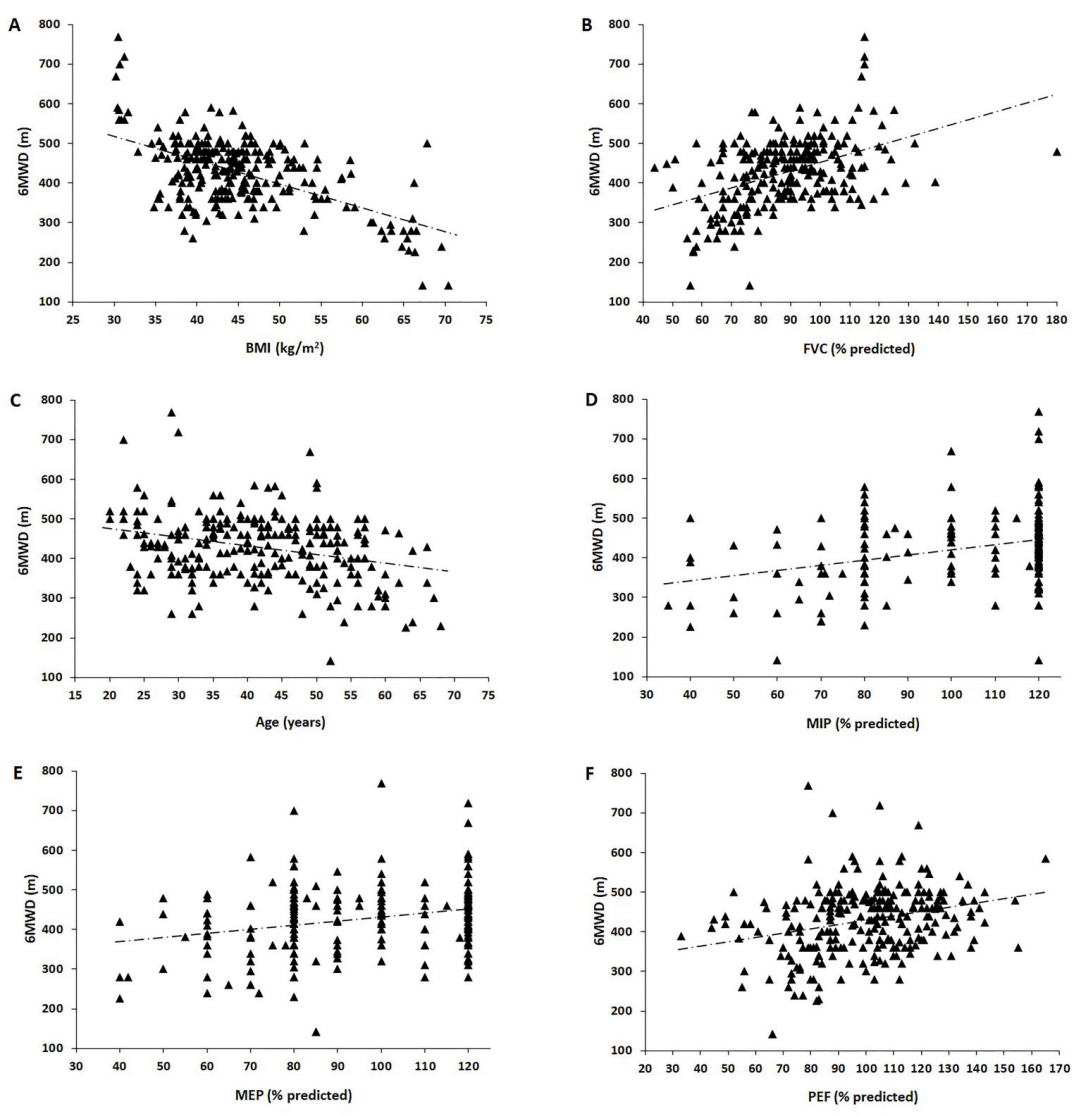
Relationship of the 6-min walk distance (6MWD) with (**A**) body mass index (BMI, r=-0.561, P<0.0001), (**B**) forced vital capacity (FVC, r=0.443, P<0.0001), (**C**) age (r=-0.281, P<0.0001), (**D**) maximal inspiratory pressure (MIP, r=0.326, P<0.0001), (**E**) maximal expiratory pressure (MEP, r=0.259, P<0.0001), and (**F**) peak expiratory flow (PEF, r=0.278, P<0.0001).

Finally, we investigated the associations between 6MWD and demographic parameters, metabolic status, and lung function (Table 2 and Figure 1). The 6MWD was positively correlated with height (r=0.319, P<0.0001), FVC (r=0.443, P<0.0001), PEF (r=0.278, P<0.0001), MIP (r=0.326, P<0.0001), and MEP (r=0.259, P<0.0001). The 6MWD correlated negatively with age (r=-0.281, P<0.0001), weight (r=-0.370, P<0.0001), and BMI (r=-0.561, P<0.0001). In the multiple linear regression, BMI and age were the independent predictive variables for 6MWD in the basic model, explaining 34% of its variability. BMI, FVC, age, and MIP were the independent predictive variables for 6MWD in the final model, explaining 41% of its variability (Table 3). Therefore, the reference equation including lung function (final model) was as follows: 6MWD (m) = 513.6 - (4.439×BMI_kg/m2_) + (1.136×FVC_%predicted_) - (1.048×age_yrs_) + (0.544 × MIP_%predicted_); R^2^=0.41 (standard error of the regression coefficient=51.8 m).

**Table 2:** Pearson's correlation coefficients for the 6-min walk distance (6MWD) with demographic parameters, nutritional status, and lung function.

Variables	6MWD	
	r	P value
Age (years)	−0.281	<0.0001
Weight (kg)	−0.370	<0.0001
Height (cm)	0.319	<0.0001
BMI (kg/m^2^)	−0.561	<0.0001
FVC (% predicted)	0.443	<0.0001
PEF (% predicted)	0.278	<0.0001
MIP (% predicted)	0.326	<0.0001
MEP (% predicted)	0.259	<0.0001

BMI: body mass index; FVC: forced vital capacity; PEF: peak expiratory flow; MIP: maximal inspiratory pressure; MEP: maximal expiratory pressure.

**Table 3:** Independent linear models for the 6-min walk distance using demographic and anthropometric parameters, nutritional status, and lung function.

Variables	B	SEB	P value	R	Adjusted R^2^	RMSE (m)	AIC
Basic model							
Constant	748.7	27.4	<0.0001				
BMI	-5.696	0.541	<0.0001	0.56	0.31	71.1	2993
Age	-1.533	0.388	0.0001	0.59	0.34	69.2	2980
Final model							
Constant	513.6	51.8	<0.0001				
BMI	-4.439	0.566	<0.0001	0.56	0.31	71.1	2993
FVC	1.136	0.251	<0.0001	0.61	0.37	68.0	2971
Age	-1.048	0.384	0.006	0.64	0.40	66.6	2961
MIP	0.544	0.204	0.008	0.65	0.41	65.8	2956

B: regression coefficient; SEB: standard error of the regression coefficient; R: cumulative correlation coefficient; R^2^: adjusted determination coefficient; RMSE: root mean square error; AIC: Akaike information criterion; BMI: body mass index; FVC: forced vital capacity; MIP: maximal inspiratory pressure.

**Figure 2:**
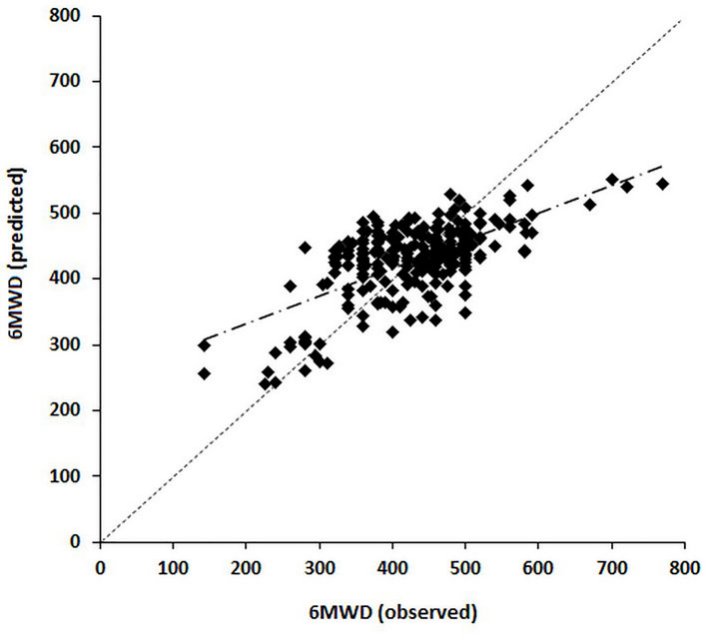
Calibration plot of the observed *vs* predicted values for the 6-min walk distance (6MWD). Pearson's correlation coefficient between the observed and predicted 6MWD was r=0.65 (P<0.0001).

**Figure 3:**
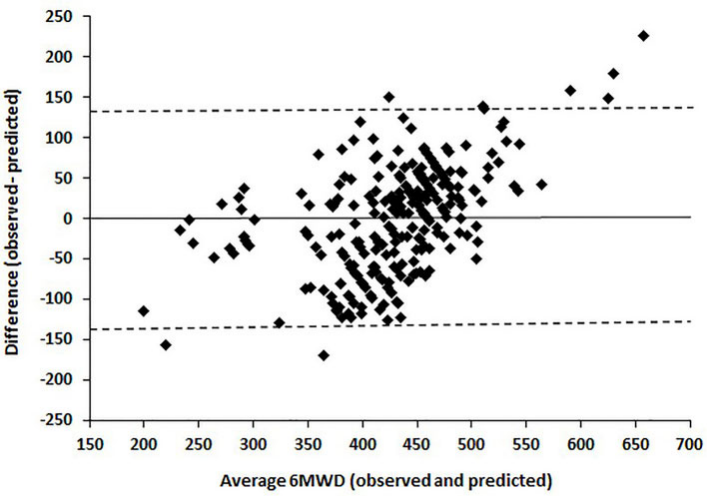
Limits of the agreement plot of the averaged values and the differences (observed - predicted values) for 6-min walk distance (6MWD). The mean difference was zero with a standard deviation of 65 m, and the corresponding 95% limits of agreement were -128 m (lower) and +128 m (upper).

Regarding the calibration of the regression model, no clearly evident relationship was detected between the differences (bias) and the mean (given by the straight line), and the fitted line had a lower slope in relation to the main diagonal (Figure 2). Based on the Bland-Altman plot (Figure 3), it was noted that the vast majority of differences were within the LoA, with a random distribution over the mean values in the range of highest concentration (350 to 500 m). However, a slight bias was observed for high and low values of distance covered. The mean difference was zero with a standard deviation of 65 m, and the corresponding 95% lower and upper agreement limits were -128 and +128 m, respectively.

## Discussion

Obesity is associated with reduced individual mobility, exacerbating sedentarism. ADLs are impaired not only because of the excessive accumulation of body fat but also because of mechanical factors that may reduce the ability to walk, the latter being a simple measure of physical function and an important component of HRQoL. We evaluated only women in the present study. There is a general consensus that women, especially shorter women, have a shorter stride length and consequently shorter distances covered in the 6MWT (4). The main findings were that in obese women, the older the age and the higher the BMI, the shorter the distance covered in the 6MWT. There was a relationship between deteriorated lung function and low 6MWD. In addition, the more severe the degree of obesity, the worse the lung function and the shorter the 6MWD. Based on these findings, we developed a predictive equation for 6MWD that considers BMI, FVC, age, and MIP, in order of importance, as independent variables.

The 6MWT is a measure of submaximal load and is a safe, practical, validated, easy-to-administer, low-cost tool to assess functional exercise capacity (6). In obese individuals, a lower functional exercise capacity is common not only because it is a weight-bearing activity but also because excess weight changes the center of gravity and increases mechanical stress on the joints and body tissues, inducing physical limitations (19). In the present study, we observed that the mean 6MWD in the total sample and the mean 6MWD in the class 3 obesity group were 428.3±85.7 m and 417.3±76.4 m, respectively. These values were slightly higher than those reported by Crispim Carvalho et al. (20) in 62 obese women aged 24-57 years but close to those observed by Vargas et al. (21) in a cohort of 67 obese individuals, including 61 (91%) women and 6 (9%) men, with a mean age of 38±10 years. Differences in the metabolic profile of the sampled population, the type and frequency of incentives, and the length of the corridor may explain the differences reported in 6MWD; therefore, these factors should be considered when interpreting the results (21).

In obese individuals, it is logical to expect BMI to be a significant predictor of 6MWD because excess weight influences gait and increases workload. In fact, our study showed that BMI was the factor that most impacted 6MWD, explaining 56% of the variance in 6MWD. Our findings are in line with those of Wooldridge et al. (6) who observed in a sample of overweight or obese American military veterans (81% men) that BMI is the variable most significantly related to 6MWD. Investigating the magnitude of the differences in walking capacity among obese and non-obese women, another study found that 75% of the variation in walking performance in obese women is explained by the combination of BMI, peak VO_2_, knee extension torque, age, and hours watching television or playing sports, with BMI alone explaining 59% of the variation (5). Interestingly, Ben Saad et al. (22) showed that when BMI is included in their final reference equation, 6MWD decreased 5.27 m for each increase of 1 unit in BMI. Obese subjects adapt to their higher body mass by decreasing walking speed and they tend to oscillate the trunk while walking and increase the distance between the ankles when stopping, to compensate for their extra body mass (23).

Obesity causes mechanical compression of the lungs, diaphragm, and chest cavity, which can lead to restrictive functional damage (10). In addition, changes in the neural control of respiration and increases in thoracic blood volume due to fat deposition in the chest promote changes in lung function parameters (10). In the present study, we observed a positive correlation between FVC and 6MWD, and FVC was also an independent predictor of 6MWD. Notably, few studies have evaluated the impact of lung function on 6MWD in non-obese individuals. Similarly, Camarri et al. (24) showed that in forward stepwise multiple regression, height and forced expiratory volume in 1 s (FEV_1_) are the only independent predictors of 6MWD in a population of overweight or obese individuals, and these variables explain 33.9% of the variance in the model. Another study showed a direct relationship between the degree of obesity and FEV_1_ and FVC in morbidly obese individuals (25). Reduced lung volume seems to have an exponential correlation with increased BMI and directly correlates with the mechanical effects produced by the deposition of fat in the chest and abdomen (26).

Structural alterations caused by obesity in the thoracoabdominal area restrict diaphragmatic mobility and rib movement, which promote changes in the dynamics of the respiratory system and reduce compliance, leading to mechanical impairment of the respiratory muscles (9,27). We observed positive correlations between 6MWD and measures of respiratory muscle strength, especially MIP. Since MIP was an independent predictor of 6MWD, we believe that its routine evaluation can assist in the analysis of thoracic cage mechanics in obese individuals. In this population, the observed dysfunction of the respiratory muscles may be partly explained by the increased resistance imposed by the presence of excess adipose tissue in the chest and abdomen, which mechanically hinders these muscles, especially the diaphragm (28).

Reference values of healthy populations with normal weight are of limited value in the obese population, since their lower muscle strength, lower effort tolerance, and higher metabolic costs during walking result in a consistently lower 6MWD (7). Some previous studies have addressed 6MWD determinants for obese subjects and proposed reference equations without considering lung function parameters (2,4,6–8). Most of these studies have incorporated age, sex, height, weight, and/or BMI into their predictive equations (2,4,6,24). The coefficients of determination in the regression analyses have ranged between 0.19 and 0.48 in the different studies, with a coefficient of determination of our adjusted final model (including lung function data) of 0.41. Although BMI is the most important independent variable in our model (and in those models of obese individuals presented in the literature), it is worth mentioning that FVC was the second variable to be included in our model as a result of the backward stepwise method. Thus, although the routine assessment of lung function in a predictive equation for 6MWD is difficult for clinical use, we think that its inclusion may contribute to a better prediction of 6MWD and an improvement in understanding the functional limitations of obese individuals.

The strength of our study is that it is the first to propose a reference equation for the 6MWD of obese Brazilian women that considered lung function as an independent variable for the predictive model. Despite the interesting results, our study has limitations. First, our results have limited generalizability because we evaluated only women. Sex has a marked influence on 6MWD, and the large imbalance between women and men in previous studies may have impacted the results (4,6,7). Second, we did not evaluate the contribution of menopause and its hormonal changes, given that menopause causes a deterioration in the function of the musculoskeletal system with reduced muscle contractility (3). Given the lack of studies on obesity and functional exercise capacity, the present study improves our understanding of the relationship between obesity and lung function and provides guidance for further research involving the 6MWT in this population. In this sense, future studies exploring 6MWT in obese women may be important in improving the model's accuracy.

In conclusion, the present study shows that in obese women, there is a correlation between the distance covered in the 6MWT and certain demographic data and metabolic status, especially age and BMI. In addition, there is an association between lung function data and 6MWD, especially FVC and MIP. These results may be useful in the rehabilitation scenario to evaluate functionality, for preoperative planning of weight reduction surgeries, to plan the intensity of physical activities, and to evaluate the success of interventions for weight loss in this population.
